# Excessive Iron Availability Caused by Disorders of Interleukin-10 and Interleukin-22 Contributes to High Altitude Polycythemia

**DOI:** 10.3389/fphys.2018.00548

**Published:** 2018-05-22

**Authors:** Yun-Sheng Liu, He Huang, Si-Min Zhou, Huai-jun Tian, Peng Li

**Affiliations:** ^1^Department of High Altitude Hygiene, College of High Altitude Military Medicine, Third Military Medical University, Chongqing, China; ^2^Department of Medical Geography, College of High Altitude Military Medicine, Third Military Medical University, Chongqing, China; ^3^Key Laboratory of High Altitude Environmental Medicine, Ministry of Education, Chongqing, China; ^4^Key Laboratory of High Altitude Physiology and High Altitude Disease, Chinese People's Liberation Army, Chongqing, China

**Keywords:** erythrocytosis, high altitude, cytokine, iron metabolism, hepcidin, inflammation, immune balance

## Abstract

**Background:** Because the pathogenesis of high altitude polycythemia (HAPC) is unclear, the aim of the present study was to explore whether abnormal iron metabolism is involved in the pathogenesis of HAPC and the possible cause.

**Methods:** We examined the serum levels of iron, total iron binding capacity, soluble transferrin receptor (sTfR), ferritin, and hepcidin as well as erythropoietin (EPO) and inflammation-related cytokines in 20 healthy volunteers at sea level, 36 healthy high-altitude migrants, and 33 patients with HAPC. Mice that were exposed to a simulated hypoxic environment at an altitude of 5,000 m for 4 weeks received exogenous iron or intervention on cytokines, and the iron-related and hematological indices of peripheral blood and bone marrow were detected. The *in vitro* effects of some cytokines on hematopoietic cells were also observed.

**Results:** Iron mobilization and utilization were enhanced in people who had lived at high altitudes for a long time. Notably, both the iron storage in ferritin and the available iron in the blood were elevated in patients with HAPC compared with the healthy high-altitude migrants. The correlation analysis indicated that the decreased hepcidin may have contributed to enhanced iron availability in HAPC, and decreased interleukin (IL)-10 and IL-22 were significantly associated with decreased hepcidin. The results of the animal experiments confirmed that a certain degree of iron redundancy may promote bone marrow erythropoiesis and peripheral red blood cell production in hypoxic mice and that decreased IL-10 and IL-22 stimulated iron mobilization during hypoxia by affecting hepcidin expression.

**Conclusion:** These data demonstrated, for the first time, that an excess of obtainable iron caused by disordered IL-10 and IL-22 was involved in the pathogenesis of some HAPC patients. The potential benefits of iron removal and immunoregulation for the prevention and treatment of HAPC deserve further research.

## Introduction

As a normal physiological compensatory response to a hypoxic environment, high-altitude (HA) migrants often experience increases in their number of red blood cells (RBCs), which improve the oxygen-carrying capacity of the blood (Windsor and Rodway, 2007). However, some migrants (~10–40%, depending on the altitude) have excessive erythropoiesis after several months or years, which results in high altitude polycythemia (HAPC), also known as chronic mountain sickness (CMS) or Monge's disease (Negi et al., 2013). Patients with HAPC experience increased blood viscosity and symptoms such as headache, dizziness, palpitations, and insomnia. Currently, there is no effective treatment method for HAPC because the pathogenesis of HAPC is complicated (Villafuerte, 2015). Although hypoxia-induced erythropoietin (EPO) is considered a major hormone that regulates erythropoiesis, the levels of EPO usually do not correlate well with erythrocyte production in many patients with HAPC (Gore et al., 2006; Painschab et al., 2015). It is necessary to explore other mechanisms that may be involved in erythrocytosis at high altitudes.

Iron has a variety of important physiological functions in the human body and is an important component of heme in hemoglobin. The increase in the number of RBCs under hypoxic conditions requires more iron utilization. Iron in the body comes from absorption of iron from the intestines (called exogenous iron), recycling of iron from the hemoglobin of damaged senescent erythrocytes and release of stored iron from the liver, muscle, and other tissues (called endogenous iron). Under HA hypoxic conditions, it is necessary to recruit more iron to provide enough materials for erythropoiesis (Cook et al., 2005; Robach et al., 2007; Gassmann and Muckenthaler, 2015). Theoretically, enhanced iron mobilization at high altitudes is controlled within a reasonable range to match the compensatory increase in RBCs to adapt to hypoxia. This raises the question of whether the status of an individual's iron metabolism (including iron storage, mobilization, and utilization) under conditions of a long-term hypoxic environment is one of the factors that limits or promotes the progression of HAPC.

Recent studies have revealed that hepcidin, which is mainly synthesized by the liver, plays a central and critical role in regulating iron homeostasis (Ganz, 2011; Deschemin et al., 2017; Sangkhae and Nemeth, 2017). Hepcidin can bind with ferroportin 1 (Fp1), a transmembrane protein in enterocytes, hepatocytes, and macrophages. After binding, Fp1 is internalized and degraded, leading to decreased exogenous intestinal iron uptake and endogenous iron release from hepatocytes and RBC phagocytic macrophages, which accurately regulates the intake and recycling of iron in the body (Nemeth et al., 2004). Studies have confirmed that disorders of hepcidin are a main contributing factor to chronic anemia in patients with infections and tumors (Reichert et al., 2017) and that hepcidin is involved in the pathological processes of hereditary hemochromatosis, thalassemia and iron deficiency anemia (Girelli et al., 2016; Daher et al., 2017).

It has been reported that exposure to high altitude hypoxic environments for several days or weeks significantly affect iron metabolism and hepcidin levels (Talbot et al., 2012; Goetze et al., 2013). It remains largely uncharacterized how iron status changes for residents who have lived at high altitudes for months or even years compared with their iron status during their early emigration period. Moreover, no literature has been reported on the features of iron metabolism and hepcidin for HAPC patients. Iron homeostasis is generally regulated by the levels of body iron load and iron demand. However, at high altitudes, many factors related to hypoxic environments may affect the regulation of iron, including chronic hypoxic stimuli (Hintze and McClung, 2011); EPO levels (Gammella et al., 2015); erythroid proliferation signals, such as growth differentiation factor 15 (Tantawy et al., 2014) and the newly discovered erythroferrone (Kautz et al., 2014); hypoxia-induced inflammatory cytokines, such as interleukin (IL)-1 and IL-6 (Hartmann et al., 2000; Lee et al., 2005; Schmidt, 2015). These may all affect the hepcidin level and the iron balance. Among the above mentioned factors, except for disordered EPO, hypoxia-induced inflammation or immune imbalance at high altitudes is a prominent problem with large individual differences in the population (Boos et al., 2016; Evstratova et al., 2016; He et al., 2016).

The present research systematically examined the iron status and relevant regulators in patients with HAPC and compared them with subjects at sea-level and with healthy high-altitude migrants. Using experiments in mice under a simulated high-altitude hypoxic environment, we also studied the effects of different levels of iron and iron-regulatory cytokines on erythrocytosis. These results revealed that the individual regulatory ability of iron metabolism was a non-negligible factor in the pathogenesis of HAPC.

## Methods

### Participants

We enrolled 20 healthy volunteers at sea level (age range 26.1 ± 3 years), 36 healthy volunteers who migrated to high altitudes (age range 26.1 ± 3.9 years), and 33 patients with HAPC who migrated to high altitudes (age range 26.5 ± 4.6 years). All participants were males of the Han nationality because of the low incidences of HAPC in females and Tibetan natives (Pei et al., 1989). The HAPC group and the high-altitude healthy group were highland settlers who lived in the Qinghai-Tibet Plateau region at altitudes of 3,850–4,200 m for 2–5 years (high-altitude healthy group, 2.77 ± 0.87 years; HAPC group, 2.88 ± 0.82 years, no significant difference). The HAPC diagnostic criteria were based on the consensus statement with regard to CMS formed by the International Society for Mountain Medicine (ISMM) at the 6th World Congress on Mountain Medicine and High Altitude Physiology (Xining, China; 2004; León-Velarde et al., 2005). HAPC patients were distinguished as having a hemoglobin concentration exceeding 210 g/L in man. Subjects with pulmonary diseases that might worsen hypoxemia were excluded from this study. The sea level control group consisted of healthy men of the Han nationality who lived in areas below 500 m and did not have a history of living at altitudes above 2,500 m. All subjects gave their written informed consent to participate in the study. The study protocol was approved by the Ethics Committee of the Third Military Medical University.

### Blood sample collection

Ethylene diamine tetraacetic acid anticoagulant tubes were used to collect 1 mL of blood. The RBC counts, hemoglobin (Hb) and hematocrit (Hct) levels were measured by pocH-100i automated hematology analyzers (Sysmex Corporation, Kobe, Japan), and the hemoglobin concentrations were verified by the cyanmethemoglobin method (Maker Technology Co., Ltd., Chengdu, Sichuan province, China). Tubes without anticoagulant were used to collect 10 mL of blood. After standing for 1 h, the serum was separated and then frozen in liquid nitrogen for the detection of iron metabolism-related indices and other cytokines within 1 week.

### Detection of serum iron metabolism indicators and related cytokines in subjects

Serum iron and total iron binding capacity (TIBC) were detected using a colorimetric method, with reagents purchased from Jiancheng Bioengineering Institute (Nanjing, Jiangsu province, China). Serum soluble transferrin receptor (sTfR), ferritin and hepcidin were detected using enzyme-linked immunosorbent assays (ELISA); detection reagents were purchased from R&D Systems (Minneapolis, MN, USA), RayBiotech (Norcross, GA, USA), and DRG International, Inc. (Springfield, NJ, USA), respectively. The EPO levels were assayed using a human ELISA kit (STEMCELL Technologies, Vancouver, BC, Canada). For serum inflammatory cytokines, the concentrations were measured using the Bio-Plex Pro^TM^ human Th17 cytokines assay (Bio-Rad Laboratories, Hercules, CA, USA) that was designed for the determination of 15 different cytokines including IL-1β, IL-4, IL-6, IL-10, IL-17A, IL-17F, IL-21, IL-22, IL-23, IL-25, IL-31, IL-33, INF-γ, sCD40L, and TNF-α, and data were acquired on a Bio-Plex HTF system.

### Mouse models with different levels of iron and cytokines under a simulated HA environment

C57BL/6 male mice weighing 20–24 g were housed in a normoxic environment or exposed to hypoxia in a hypobaric chamber (equivalent to altitude 5,000 m). For one group of the hypoxic mice, saline or iron dextran solution (Pharmacosmos company, Holbaek, Denmark) of various concentrations (high, 100 mg/kg bw; medium, 50 mg/kg bw; low, 25 mg/kg bw) was injected once every other day. Another group of mice received recombinant murine (rm) IL-10 (0.2 mg/kg bw), rm IL-22 (0.2 mg/kg bw), anti-IL-10 (10 mg/kg bw), or anti-IL-22 (10 mg/kg bw) via intraperitoneal injection once every other day. The recombinant cytokines were purchased from PeproTech (Rocky Hill, NJ, USA), and neutralizing antibodies were purchased from R&D Systems (Minneapolis, MN, USA). After 4 weeks, the mice were anesthetized, and blood was collected through the fossa orbitalis. The numbers of RBCs and the concentrations of hemoglobin and iron in the murine blood were detected using the same methods as human blood, which were described above. To detect bone marrow (BM) erythropoiesis, BM cells were collected from the femurs and tibias by flushing with PBS solution. All animal procedures were performed according to the Guidelines for the Use and Care of Laboratory Animals established by the Third Military Medical University.

### Detection of bone marrow erythroid hematopoietic growth in mice

Bone marrow cells flushed out from the hind limbs of mice were filtered through a mesh. The single-cell suspensions of 2 × 10^6^ cells in PBS with 2% fetal calf serum were blocked with CD16/32 antiobdy (1 μg/ml; eBioscience, San Diego, CA, USA) on ice for 10 min, and then stained with PE-anti-mouse CD71 (1 μg/ml; eBioscience) and FITC-anti-mouse Ter119 (1 μg/ml; eBioscience) for 30 min. Dead cells and nonspecific signals were excluded by propidium iodide (Sigma, St. Louis, MO, USA) staining and appropriate isotype controls (eBioscience). A BD FACS Calibur flow cytometer (BD Biosciences, Franklin Lakes, NJ, USA) was used to detect the percentage of erythroid precursor cells at different maturation stages. For the colony formation units counting, the another part of the BM cells were incubated in ACK lysis buffer for 10 min at room temperature to obtain the nucleated cells. The 2 × 10^4^ BM nucleated cells were embedded in 1 ml of Stemcell M3434 or M3334 semi-solid medium (StemCell Technologies, Vancouver, Canada) at 37°C, 5% CO_2_ for 10 or 2 days, and then colonies numbers of burst-forming units-erythroid (BFU-E) and colony formation units-erythroid (CFU-E) were observed, respectively.

### Detection of serum hepcidin and hepatic hepcidin mRNA of mice

Serum hepcidin concentrations of mice were determined with the mouse hepcidin 25 ELISA kit (Abbexa Ltd., Cambridge, UK). The total RNA from the liver tissue of the mice was extracted using an RNAiso kit (TaKaRa, Dalian, China). Next, the Takara PrimeScript RT application kit was used for the reverse transcription step. The expression of hepcidin mRNA was measured by a fluorescence quantitative PCR method, and the Power SYBR Green PCR Master Mix Kit was purchased from Applied Biosystems (Foster City, CA, USA). The mouse hepcidin (Mu Hamp1) amplification primers were forward 5′-CCAACTTCCCCATCTGCATCTTCTGC-3′ and reverse 5′-GGCAGACATTGCGATACCAAT-3′. The relative mRNA expression was normalized to GAPDH mRNA, and the results were calculated using the 2^−ΔΔCt^ method.

### *In-vitro* culture of bone marrow hematopoietic progenitor cells

BM nucleated cells collected from three C57BL/6J mice were lineage (Lin) depleted with the IMag^TM^ Mouse HSPCs Enrichment Set (BD Biosciences, San Jose, CA). Then, the 2 × 10^4^ BM Lin^−^ hematopoietic progenitor cells were suspended in 1 ml StemSpan serum-free expansion medium (StemCell Technologies, Vancouver, Canada) with the presence of 10% fetal calf serum, 10 ng/mL rmIL-3, 50 ng/mL rm stem cell factor, and 5 U/mL recombinant human EPO. The rmIL-10 and rmIL-22 were selectively added to the culture system with different concentrations. A part of cells were harvested after 3 days of culture, and the numbers of BFU-E and CFU-E were determined using StemCell Methocult M3434 or M3334 medium according to the methods mentioned above. For another part of cells, after an additional 4 days of culture (a total of 7 days), erythroid cells were analyzed by Ter119/CD71-stained FCM analysis.

### Statistical analysis

Data are presented as the mean ± standard deviation or the median (25, 75% percentiles). For data with a normal distribution, differences between the groups were examined for statistical significance using one-way analysis of variance followed by a least significant difference test. For data with non-normal distributions, multiple groups were compared using the non-parametric rank sum test. Correlations between the indices were analyzed using the Pearson or Spearman analyses. All statistical analyses were performed using SPSS 13.0. Statistical significance was reported when *P* < 0.05.

## Results

### Vigorous erythropoiesis at high altitude is accompanied by enhanced iron mobilization, utilization, and storage

Serum iron, TIBC, and sTfR reflect the available iron, the capacity of iron transportation and the velocity of erythrocytes to utilize iron, respectively. Table 1 presents the differences in these iron indicators among the three groups. There was a significant increase in serum iron and sTfR in the healthy HA migrants compared with the sea-level residents, reflecting a remarkable increase in iron mobilization and utilization. Compared with the HA healthy men at the same elevation, HAPC patients with excessive erythrocytosis exhibited further increases in serum iron and sTfR as well as TIBC related to iron transportation, revealing sufficient iron availability. Further analysis among the 69 HA migrants (including 36 healthy individuals and 33 patients with HAPC) showed that serum available iron was positively associated with hemoglobin content, which is an indicator reflecting the degree of erythrocytosis.

**Table 1:** Serum iron status indicators among participants in this study ($\bar{X}\pm$ S).

	**Sea level group (*n* = 20)**	**HA healthy group (*n* = 36)**	**HAPC patients group (*n* = 33)**	**Correlation with Hb in HA subjects (*n* = 69)**
RBC (× 10^12^/L)	4.71 ± 0.23	5.89 ± 0.33^**^	6.62 ± 0.41^▴▴^	
Hb (g/L)	143.3 ± 13.4	186.7 ± 9.8^**^	223 ± 10.2^▴▴^	
Hct (%)	49.70 ± 3.15	56.70 ± 5.55^**^	66.20 ± 5.45^▴▴^	
Iron (μmol/L)	95.45 ± 8.66	108.61 ± 15.89^**^	121.7 ± 12.96^▴▴^	r = 0.340; *P* = 0.004
TIBC (nmol/L)	312.6 ± 46	332.4 ± 48.7	371.5 ± 76.3^▴▴^	r = 0.062; *P* = 0.612
sTfR (nmol/L)	18.85 ± 3.1	26.09 ± 4.8^**^	34.92 ± 6.75^▴▴^	r = 0.717; *P* = 0.000
Ferritin (ng/ml)	149 ± 30.8	139.1 ± 59.9	187 ± 65.9^▴▴^	r = 0.146; *P* = 0.106

** *P < 0.01; significantly different from the sea level group*.

▴▴ *P < 0.01; significantly different from the HA healthy group*.

*The r and P represent the Pearson correlation coefficient and the two-tailed P-value, respectively*.

Serum ferritin concentrations reflect the amount of storage iron. As shown in Table 1, there was no difference in the serum ferritin levels between the HA healthy group and the sea level group. But on average, the ferritin levels of HAPC patients were markedly higher than those of the healthy HA migrants, which indicated that redundant iron from damaged RBCs accumulated in some patients with HAPC.

### Hepcidin regulated iron mobilizing capacity at high altitudes

**Figure 1:**
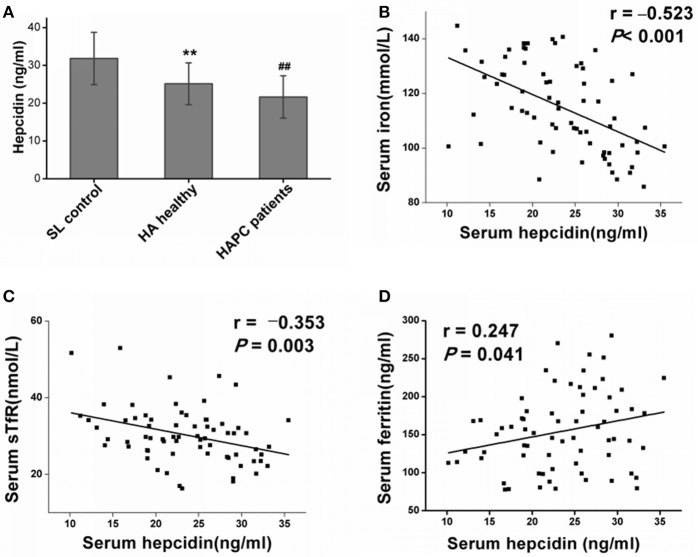
High-altitude hypoxia affects hepcidin levels and the association with iron mobilization and utilization. **(A)** Individual data for serum hepcidin levels from the sea level (SL) control group, the high-altitude (HA) healthy group and the HAPC patients group. ^**^P < 0.01; significantly different from the sea level group. ^##^*P* < 0.01; significantly different from the HA healthy group. **(B,C)** Serum hepcidin levels were negatively correlated with iron and sTfR levels in all the high-altitude migrants (including healthy people and patients with HAPC). **(D)** A positive correlation between hepcidin and ferritin levels was observed in the HA migrants. Pearson's correlation coefficient and *P*-values for each correlational analysis are given.

As the key molecule that regulates iron metabolism, serum hepcidin levels were significantly reduced in the HA healthy migrants in contrast with the sea level control group, which matched with a reasonable compensatory increase in iron mobilization for RBC production during hypoxia. Furthermore, the serum hepcidin concentrations were much lower in the HAPC patient group than they were in the HA healthy group (Figure 1A). There was a strong inverse relationship between hepcidin and serum iron (Figure 1B) as well as a negative association between serum hepcidin and sTfR levels (Figure 1C) among all of the HA migrants, which suggested that the lower serum hepcidin concentrations were linked to higher iron mobilization and utilization for erythropoiesis. In addition, hepcidin correlated positively with serum ferritin levels (Figure 1D), which suggested that downregulated hepcidin led to increased iron release from the iron storage cells at high altitudes. To summarize, hepcidin disorders played a dominant role in the excessive iron availability in HAPC.

### Decreased IL-10 and IL-22 in HAPC are associated with decreased hepcidin levels

**Figure 2:**
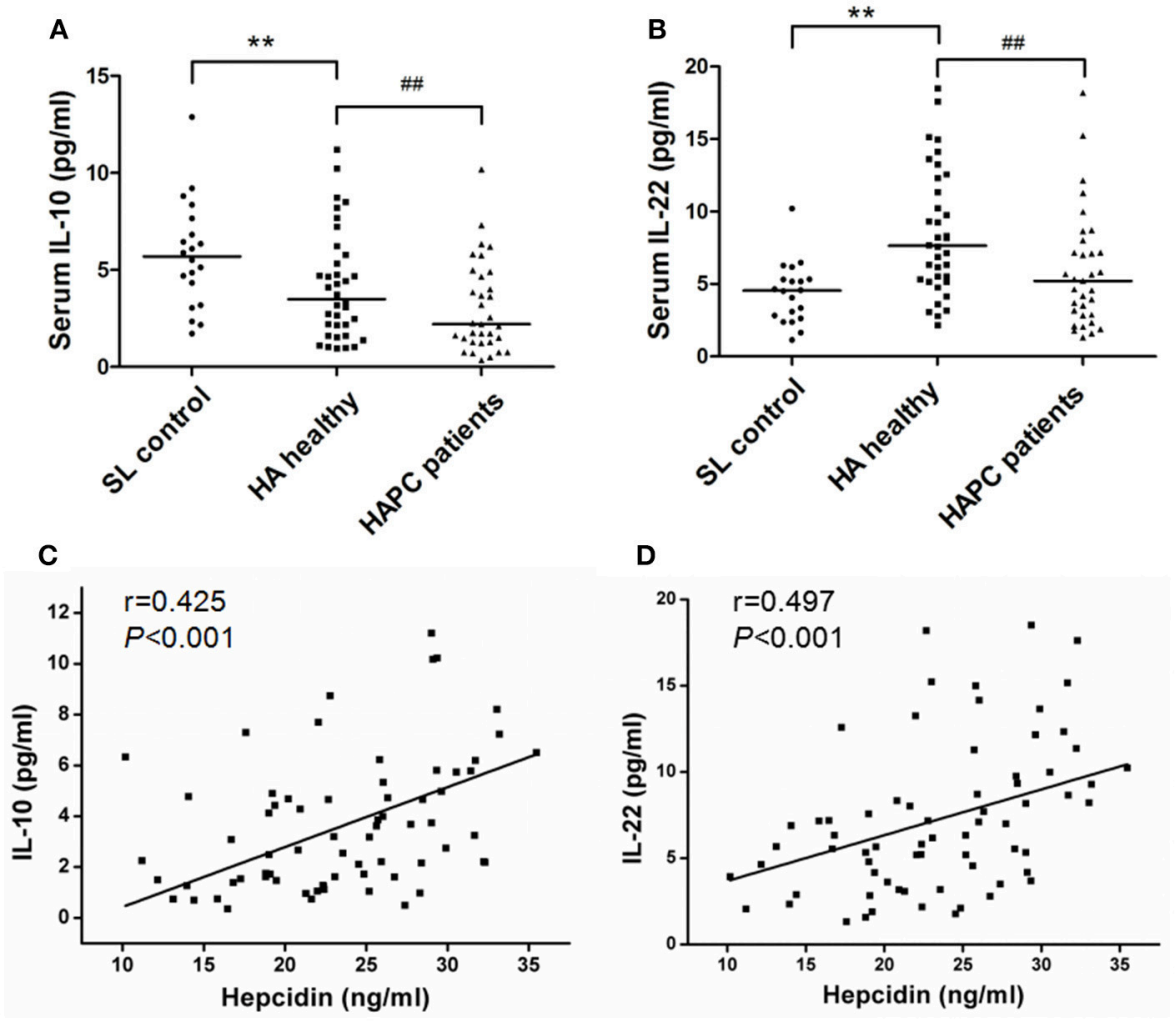
Difference in IL-10 and IL-22 production between healthy HA migrants and patients with HAPC and the association with hepcidin levels. **(A,B)** Comparison of serum IL-10 and IL-22 levels among the participants of each group. ^**^P < 0.01; significantly different from the sea level group. ^##^*P* < 0.01; significantly different from the HA healthy group. **(C,D)** IL-10 and IL-22 levels were positively correlated with hepcidin concentrations in all of the HA migrants. Spearman's correlation coefficient (r) and *P*-values are labeled.

The results obtained from the ELISA showed an obvious elevation of serum EPO, IL-1β, and IL-6 levels in HA migrants compared with sea-level residents, but no substantial difference was found between HAPC and HA healthy subjects (Table 2). Under HA conditions, IL-10 decreased and IL-22 increased in healthy people in contrast to people at sea level. It was noteworthy that HAPC patients were characterized by markedly decreased circulating IL-10 and IL-22 concentrations compared to the HA healthy group (Figures 2A,B). As immune cytokines associated with IL-10 and IL-22, Th17 cytokines IL-17A, and IL-21 were significantly elevated in HAPC patients (Table 2). Interestingly, both IL-10 and IL-22 were positively correlated with serum hepcidin content (Figures 2C,D). The subjects with lower IL-10 and IL-22 levels also had higher serum iron and hemoglobin levels (Table 2). This raised the possibility that IL-10 and IL-22 may play important roles in regulating hepcidin and iron availability during hypoxic conditions at high altitudes.

**Table 2:** Serum levels of potential hepcidin regulators for the subjects in the three groups.

	**Sea level group (*n* = 20)**	**HA healthy group (*n* = 36)**	**HAPC patients group (*n* = 33)**	**Correlation with serum hepcidin in HA subjects (*n* = 69)**	**Correlation with serum iron in HA subjects (*n* = 69)**	**Correlation with Hb contents in HA subjects (*n* = 69)**
EPO	24.4	35.4 ^**^	37.7	r = −0.163	r = 0.046	r = 0.197
(mIU/ml)	(20.9-27.7)	(28.3-41.6)	(27.2-47.1)	*P* = 0.182	*P* = 0.705	*P* = 0.104
IL-1β	0.05	0.12 ^**^	0.11	r = 0.141,	r = −0.113	r = 0.170
(pg/ml)	(0.03-0.12)	(0.04-0.22)	(0.03-0.22)	*P* = 0.248	*P* = 0.355	*P* = 0.163
IL-6	0.20	0.56^**^	1.39	r = −0.130	r = −0.201	r = 0.241
(pg/ml)	(0.10-0.36)	(0.31-4.79)	(0.29-3.26)	*P* = 0.286	*P* = 0.099	*P* = 0.046
IL-10	5.71	4.2 ^**^	2.21^▴▴^	r = 0.425	r = −0.317	r = −0.293
(pg/ml)	(4.04-6.81)	(2.41-5.56)	(1.28-4.66)	*P* = 0.000	*P* = 0.008	*P* = 0.015
IL-22	4.54	6.61^**^	5.21 ^▴▴^	r = 0.497	r = −0.251	r = −0.401
(pg/ml)	(2.78-5.32)	(5.08-8.69)	(2.88-7.18)	*P* = 0.000	*P* = 0.038	*P* = 0.001
IL-17A	1.06	1.96^**^	5.3^▴▴^	r = −0.176	r = 0.198	r = 0.242
(pg/ml)	(0.57-2.08)	(0.82-4.84)	(1.7-8.15)	*P* = 0.148	*P* = 0.104	*P* = 0.045
IL-21	10.85	16.28^**^	29.84^▴▴^	r = −0.160	r = 0.194	r = 0.224
(pg/ml)	(6.14-21.02)	(10.85-35.27)	(18.99-118.52)	*P* = 0.190	*P* = 0.111	*P* = 0.064

*The results are presented as the median (25th and 75th percentiles)*.

** *P < 0.01; significantly different from the sea level group*.

▴▴ P < 0.01; significantly different from the HA healthy group

*The r and P represent the Spearman correlation coefficient and the two-tailed P value, respectively*.

### Effects of increased iron availability on RBC production in hypoxic mice

**Figure 3:**
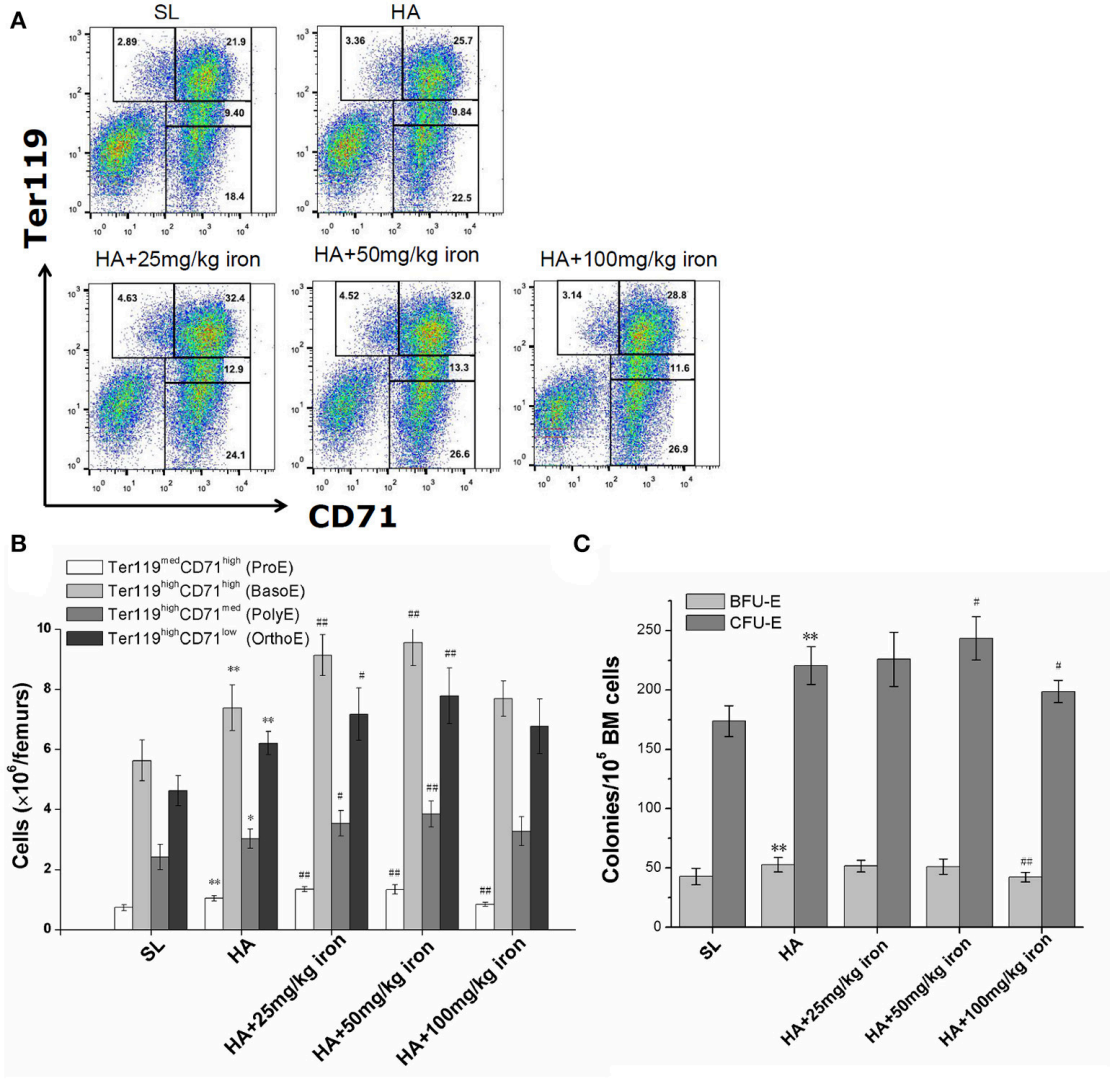
Effects of different levels of iron load on erythroid hematopoiesis in the bone marrow. Mice were subcutaneously administered iron dextran at different concentrations during a 4-week period of hypoxic exposure. Then, the BM cells were analyzed. **(A)** Representative FCM staining profiles of the BM Ter119^med^CD71^high^ proerythroblasts (ProE), Ter119^high^CD71^high^ basophilic erythroblasts (BasoE), Ter119^high^CD71^med^ polychromatophilic erythroblasts (PolyE) and Ter119^high^CD71^low^ orthochromatic erythroblasts (OrthoE) in each group. The number in each quadrant indicates the percentage in all BM cells. **(B)** The numbers of erythroid precursors at all stages in two femurs were calculated based on the total number of cells and the percentages of subgroups (*n* = 4). **(C)** The BFU-E and CFU-E in BM cells of two femurs were analyzed for each group (*n* = 6). The number of colonies of a mice were the means of triplicate cultures. All the above data were representative of two independent experiments giving similar results. ^**^P < 0.01; vs. the control group at sea level, #*P* < 0.05; ##*P* < 0.01; vs. the control group at high altitude.

To explore whether enhanced iron availability at high altitudes contributed to erythropoiesis or was only an accompanying side effect of hypoxia-induced polycythemia, mice in a high altitude environment were administered exogenous iron dextran. Serum iron detection verified that mild, moderate, and massive increases in iron availability were successfully established in the animal models (Table 3). As shown in Table 3, under 5,000 m high-altitude hypoxic conditions, low (25 mg/kg) and moderate (50 mg/kg) doses of iron dextran, which were continuously injected every other day for 4 weeks, significantly increased the RBC counts and hemoglobin concentrations in peripheral blood, but a high dose (100 mg/kg) of iron dextran had an inhibitory effect on RBC and hemoglobin production. Similar to the peripheral blood results, low and moderate doses of iron supplementation, but not high doses, had an apparent stimulatory effect on BM erythropoiesis, which was expressed as a substantial increase in absolute numbers of CD71/Ter119 distinguished erythroid cells at different stages, including Ter119^med^CD71^high^ proerythroblasts (ProE), Ter119^high^CD71^high^ basophilic erythroblasts (BasoE), Ter119^high^CD71^med^ polychromatophilic erythroblasts (PolyE) and Ter119^high^CD71^low^ orthochromatic erythroblasts (OrthoE) (Figures 3A,B). At the upstream of erythroblast, the moderate doses of iron supplementation also resulted in an increase in the number of CFU-E, reflecting the expansion of later erythroid progenitor cells (Figure 3C).

**Table 3:** Iron status and erythrocyte parameters of mice with different levels of iron and IL-10/IL-22 in high-altitude (HA) environments ($\bar{X}\pm$ S, *n* = 6).

	**Iron (μmol/L)**	**RBC count (× 10^12^/L)**	**Hb (g/L)**
Sea level control	13.42 ± 0.7	8.17 ± 0.21	152.6 ± 5.7
HA control	16.54 ± 0.61^**^	11.08 ± 0.61^**^	188.9 ± 5.413^**^
HA + iron (25 mg/kg)	18.67 ± 1.08^▴▴^	11.88 ± 0.39^▴▴^	195.7 ± 5^▴^
HA + iron (50 mg/kg)	23.64 ± 1.09^▴▴^	12.23 ± 0.4^▴▴^	202.5 ± 5.7^▴▴^
HA + iron (100 mg/kg)	27.28 ± 1.52^▴▴^	10.35 ± 0.57^▴▴^	181.2 ± 3.9^▴^
HA + IL-10	12.67 ± 0.89^▴▴^	9.60 ± 0.39^▴▴^	173 ± 5.2^▴▴^
HA + IL-22	14.98 ± 0.84^▴▴^	10.22 ± 0.37^▴▴^	176 ± 5.1^▴▴^
HA + anti-IL-10	22.16 ± 1.1^▴▴^	12.02 ± 0.41^▴▴^	197.4 ± 3.8^▴▴^
HA + anti-IL-22	20.38 ± 0.58^▴▴^	11.78 ± 0.45^▴▴^	195.1 ± 4.2^▴^

*Data are from a representative mice experiment performed twice with similar results*.

** *P < 0.01; significantly different from the saline control group at sea level*.

▴ *P < 0.05*,

▴▴ *P < 0.01; significantly different from the saline control group at high altitude*.

### Effects of IL-10 and IL-22 on hematopoiesis in hypoxic mice

**Figure 4:**
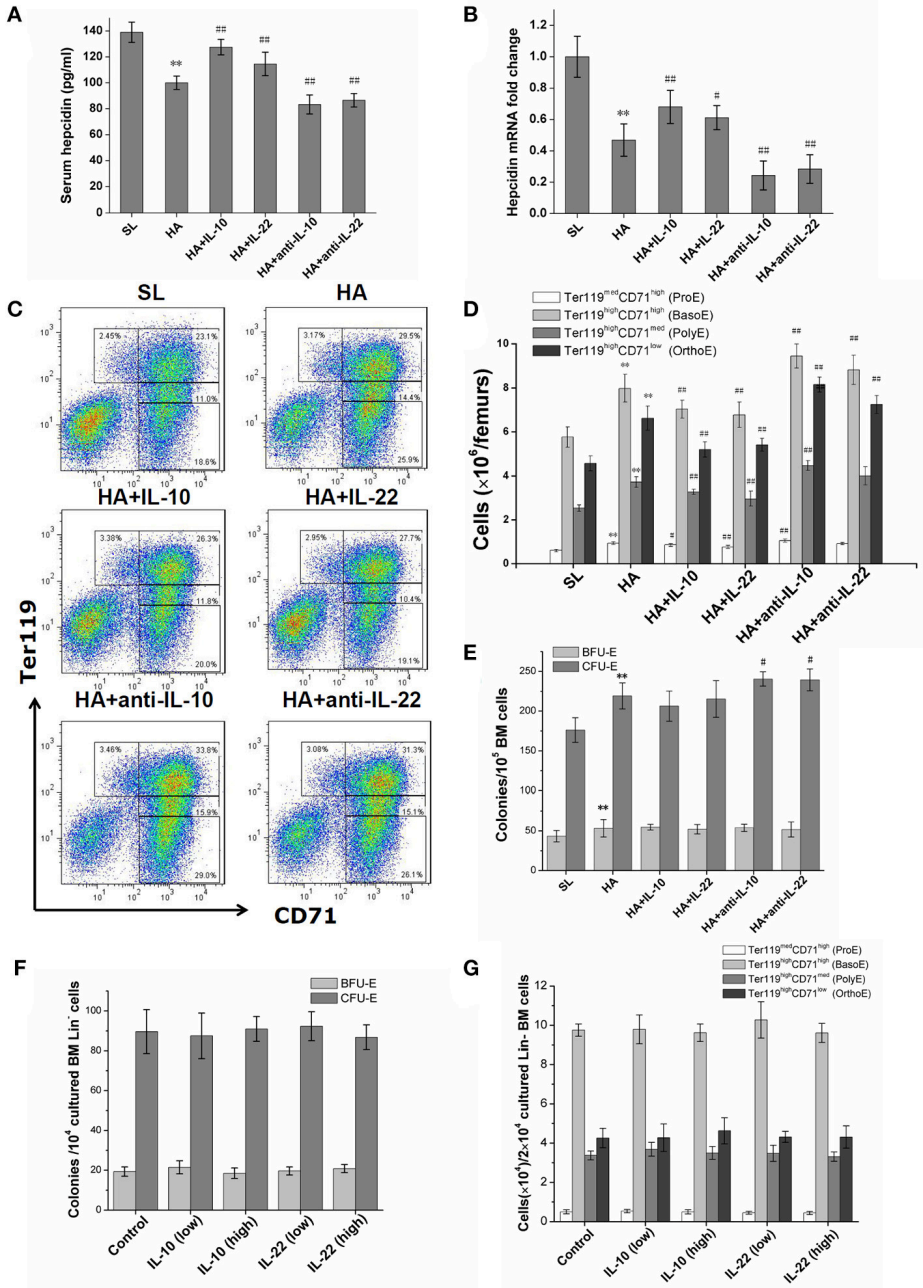
Effects of exogenous supplementation or inhibition of IL-10 and IL-22 on hepcidin expression and bone marrow erythroid precursors at high altitude. **(A)** The serum hepcidin concentrations of mice were determined by ELISA in repeated wells. **(B)** The expression of hepcidin mRNA in the livers of mice was assessed by RT-PCR and was performed in duplicate (*n* = 6). **(C,D)** The numbers of BM erythroblasts at different stages were determined by CD71/Ter119 staining and flow cytometry analysis (*n* = 6). **(E)** Erythroid progenitors in BM cells were observed by BFU-E and CFU-E colony formation assays (*n* = 6). All mice data were from one experiment representative of two independent experiments with similar results. ^**^P < 0.01; vs. the control group at sea level, ^#^*P* < 0.05; ^##^*P* < 0.01; vs. the control group at high altitude. **(F,G)** BM Lin- cells of mice were cultured in liquid medium containing erythroid growth cytokines with or without rmIL-10 or rmIL-22. The BFU-E and CFU-E clonogenicities of output cells were analyzed after 3 days of culture, and the generation of erythroblasts were observed by CD71/TER119-staining after 7 days of culture. The means ± SD of triplicates done in one experiment representative of three independent experiments giving similar results are shown.

For mice under hypoxic conditions, the injection of IL-10 and IL-22 was associated with significantly increased serum hepcidin levels and enhanced hepcidin mRNA expression in liver (Figures 4A,B), and decreased serum iron, RBC counts and hemoglobin concentrations in the peripheral blood (Table 3) and a decreased number of CD71/Ter119 stained erythroid precursor cells in the BM (Figures 4C,D). In contrast, both anti-IL-10 and anti-IL-22 neutralizing antibodies inhibited hepcidin expression markedly (Figures 4A,B) and mobilized more iron into the blood. Erythroid hematopoiesis was also enhanced, as reflected by increased RBC counts and elevated hemoglobin concentrations (Table 3). Anti-IL-10 or anti-IL-22 treatment also led to a larger population of the CD71+ or TER119+ erythroid precursors as well as more erythroid progenitors represented by CFU-E in the BM (Figures 4C–E). *In vitro* culture of bone marrow hematopoietic progenitors showed that neither IL-10 or IL-22 had direct effect on proliferation or erythroid differentiation of progenitors in a wider range of common effect concentrations (Figure 4F). These two cytokines also did not change the maturation and production of CD71/Ter119-stained erythroid precursor cells *in vitro* (Figure 4G).

## Discussion

Our study found, for the first time, that increased iron availability was a notable feature of patients with HAPC and was associated with disordered IL-10 and IL-22. We performed experiments in mice and demonstrated that excessive obtainable iron can promote erythroid hematopoiesis, and the diminished hepcidin resulting from decreased IL-10 and IL-22 was an important cause of excess iron supply. These results from human and murine studies suggest that an imbalance in immune-inflammatory cytokines participate in promoting excessive RBC production in some patients with HAPC. With the increase in the RBC counts and hemoglobin levels among the high altitude migrants, serum sTfR released by the erythropoietic precursors significantly increased because of a higher speed of iron use (Beguin, 2003). Meanwhile, total serum iron and TIBC were elevated in the HA migrants compared with the sea-level residents, which revealed that more iron was prepared due to advancing erythropoietic demands (Northrop-Clewes and Thurnham, 2013). In patients with HAPC, the vigorous production of RBCs resulted in further enhanced iron utilization compared with healthy HA migrants. In parallel, we observed a higher level of ferritin in patients with HAPC, which reflects excessive iron storage from the continuous destruction of aging RBCs (Salvin et al., 2014). It is remarkable that the serum iron levels were much higher in patients with HAPC due to increases in iron mobilization from exogenous absorption and endogenous reuse. The simultaneous increase in iron storage and iron mobilization in HAPC patients suggests that the massive amount of iron coming from damaged RBCs constantly repeats the cycle of steps involved in storage and reuse. In addition, iron availability was significantly associated with the severity of HAPC. This result has not been reported in previous studies.

To clarify whether easy access to iron is a risk factor for or a stimulus of HAPC, we injected mice with different concentrations of iron, establishing mouse models with different levels of iron availability under high altitude hypoxic conditions, and then observed the effects on erythropoiesis. Similar to previous studies (Hartmann et al., 2013; Lu et al., 2013), we found that a high concentration of iron can inhibit hematopoiesis, particularly the BFU-E and CFU-E progenitor cells and Ter119^med^CD71^high^ proerythroblasts, indicating iron toxicity to erythroid precursors of earlier differentiation stage. However, we also found that low and moderate levels of extra iron supply, as observed in HAPC patients, can promote erythroid precursors formation in the BM, especially the cells after CFU-E stage which begin to express the transferrin receptor (Suzuki et al., 2003). Unlike the BM of patients with aplastic anemia and myelodysplastic syndrome, which is more sensitive to iron toxicity as a result of poor BM status (Della Porta et al., 2015), the BM tissues of hypoxia-induced HAPC patients were not usually damaged. Thus, excessive iron within a certain range could be transformed into a favorable factor to promote erythroid hematopoiesis in HAPC, which contributed to the worsening of uncontrolled erythrocytosis and was related to the severity of HAPC.

Consistent with the results on short-term HA travelers published by others (Piperno et al., 2011), our results showed that hepcidin levels remained continuously low during chronic hypoxic exposure in long-term HA residents. Crucially, we found a remarkable decrease in hepcidin levels in patients with HAPC compared with healthy migrants who lived at the same altitude. Decreased hepcidin may be involved in the occurrence of excessive iron mobilization and subsequent runaway polycythemia among some HAPC patients, and this hypothesis is supported by the correlation analysis between the hepcidin levels and both serum iron concentrations and hemoglobin contents. The decrease in hepcidin is beneficial to the body if it is moderate and compatible with the physiological iron demand at high altitudes. However, greater decreases in hepcidin in HAPC may result in excessive iron absorption and the reuse and release of stored iron to exceed the iron demands of the body. It can be speculated that those without HAPC have controlled iron availability to synthesize hemoglobin. To a certain extent, iron deficiency limits the rate of erythrocyte formation and the development of HAPC. In contrast, it is easier for HAPC patients to obtain iron than others, particularly through the reuse of iron from damaged aging RBCs, as raw material for generating RBCs.

As the leading hormone that regulates body iron metabolism under high altitude hypoxic conditions, hepcidin was regulated in response to a series of signaling pathways related to hypoxic exposure, including signals involved in increased iron demand, such as transferrin (Bartnikas et al., 2011); signals related to enhanced erythropoiesis, such as GDF-15 (Ronzoni et al., 2015); and erythroid growth factors, such as EPO (Ribeiro et al., 2016). EPO and inflammation are the two most prominent features under high altitude conditions that may influence adaptation to the hypoxic environment (Lemos Vde et al., 2013). Our results showed that although EPO, IL-6, and IL-1β were easily increased in HA migrants, the possibility that these cytokines induced hepcidin downregulation was ruled out because no substantial difference was observed between the HA subjects with and without HAPC.

The crucial findings of this study were the roles of IL-10 and IL-22 in influencing the incidence and severity of HAPC in a hypoxic HA environment. Hypoxia usually leads to oxidative stress and inflammation, and some HA migrants could not launch an effective anti-inflammatory defense, which is involved in the pathogenesis of acute and chronic HA diseases. IL-10 is an anti-inflammatory cytokine that is produced by monocytes, type 2 T helper cells and CD4+, CD25+ and Foxp3+ regulatory T cells (Tregs), and lower IL-10 is associated with susceptibility to acute mountain sickness (Liu et al., 2017). IL-22 is produced by activated NK and T cells, and its role involves innate immune responses and Th17 function. Although IL-22 has a pro-inflammatory effect in the acute inflammatory response, it often plays a protective role in the process of chronic inflammation (Wolk et al., 2011). Recent studies have suggested that hypoxia may inhibit the expression of IL-22 and its activity (Budda et al., 2016, 2017). Our observations suggested that the progression of HAPC was accompanied by a reduction in these two protective cytokines and an a higher inflammatory susceptibility, which were also supported by the detection result of other pro-inflammatory cytokines such as higher IL-17A and IL-21 in HAPC subjects. We speculate that the immune balance of Treg and Th17 in HAPC patients may be broken, showing a trend toward Th17 polarization and a predominance of proinflammatory power.

We excluded the possibility that IL-10 and IL-22 affected erythroid production through direct stimulation of progenitors and erythroblasts. The decrease of these two cytokines should affect the erythroid hematopoiesis in an indirect way. IL-10 and IL-22 have been shown to trigger the expression of hepcidin in previous studies (Smith et al., 2013; Chang et al., 2014; Huang et al., 2014; Wallace and Subramaniam, 2015). IL-10 and IL-22 share common receptors, and both can activate the STAT3 signaling pathway of the target cells; in turn, STAT3 can upregulate the transcription of hepcidin in various cells (Lu et al., 2016; Kong et al., 2018). During the inflammatory process caused by infections, elevated IL-22 can limit iron availability to bacteria through inducing hepcidin production, which leads to suppression of bacterial growth (Sakamoto et al., 2017). In contrast, the immune activation caused by HA hypoxia is non-pathogenic, and IL-10 and IL-22 have different expression patterns and biological effects. Although hypoxia itself inhibits the production of hepcidin directly or indirectly, our results from the mice confirmed that reduced IL-10 and IL-22 were beneficial to the maintenance of hepcidin at a lower level during long-term hypoxia. This explains why HAPC patients with lower levels of IL-10 and IL-22 had lower hepcidin levels and more available iron for RBC production.

## Conclusion

The present study confirms that the individual control of iron metabolism is not well matched with a reasonable compensatory increase in RBCs in some HAPC patients. The abnormal hepcidin caused by dysregulation of IL-10 and IL-22 is involved in the progression of HAPC. Our data suggest a new theory of pathogenesis and provide some prevention and treatment ideas for HAPC. For those HAPC patients with excessive iron load, the actual effects of iron removal therapy and immunomodulatory treatment can be explored in the future.

## Author contributions

PL designed the study and drafted the report; Y-SL contributed to samples collection and detection; HH and S-MZ contributed the laboratory experiments; HT did the statistical analysis. All authors revised the manuscript critically for important intellectual content and approved the final manuscript.

### Conflict of interest statement

The authors declare that the research was conducted in the absence of any commercial or financial relationships that could be construed as a potential conflict of interest. The reviewer EMP and handling Editor declared their shared affiliation.

## Abbreviations

BasoE: basophilic erythroblasts
BM: bone marrow
BFU-E: burst-forming units-erythroid
CFU-E: colony formation units-erythroid
ELISA: enzyme-linked immunosorbent assays
EPO: erythropoietin
Fp1: ferroportin 1
HA: high-altitude
HAPC: high altitude polycythemia
Hb: hemoglobin
Hct: hematocrit
IL: interleukin
OrthoE: orthochromatic erythroblasts
PolyE: polychromatophilic erythroblasts
ProE: proerythroblasts
RBCs: red blood cells
rm: recombinant murine
sTfR: soluble transferrin receptor
TIBC: total iron binding capacity
Tregs: regulatory T cells.
